# Correlation between glycemic control and left ventricular diastolic dysfunction in type 2 diabetes

**DOI:** 10.6026/973206300214084

**Published:** 2025-11-15

**Authors:** Aparnna Unnikrishnan Nair, Sorabh Sharma, Asma Aara Younus, Jonathan Roy Varghese, Shanmukha Koppolu, Sharik Mehraj Patloo

**Affiliations:** 1 Department of Internal Medicine, Altnagelvin Hospital, Western Health and Social Care Trust, Londonderry, Northern Ireland, UK; 2 Department of Internal Medicine, University of Arizona, Tucson, Arizona, USA; 3 Department of Infectious Disease, AI Hada Armed Forces Hospital, Taif, KSA, Saudi Arabia; 4 Department of Internal Medicine, Triunelveli Medical College, Tirunelveli, Tamil Nadu, India; 5 Department of Trauma and Orthopaedics, Royal London Hospital, Barts NHS Trust, London, England/ UK; 6 Department of General Internal Medicine, SKIMS Medical College, Srinagar, Jammu Kashmir, India

**Keywords:** Type 2 diabetes mellitus, glycated hemoglobin, diastolic dysfunction, left ventricular function, echocardiography

## Abstract

Type 2 diabetes mellitus (T2DM) has been linked to higher cardiovascular morbidity and left ventricular diastolic dysfunction (LVDD). The potential influence of the severity of cardiac dysfunction T2DM patients may depend on diabetes control measures and HbA1c concentration levels. Therefore, it is of interest to determine the relationship between HbA1c and LVDD in patients with type 2 DM by measurement echocardiographic parameters, along diastolic dysfunction and poor glycemic control as indications of the presence of an association. Early cardiac screening interventions may lead to lower long-term cardiovascular morbidity in population studies of poorly controlled diabetic patients.

## Background:

Type 2 Diabetes Melitus (T2DM) is an international burden because it is largely associated with morbidity and mortality due to cardiovascular disease [1]. Left ventricular diastolic dysfunction (LVDD), can present as part of diabetic cardiomyopathy and can occur even in the absence of clinically relevant coronary artery disease or hypertension [2]. Pathophysiologically, diastolic dysfunction is explained by abnormal calcium handling, microvascular dysfunction and myocardial fibrosis [3]. Many macrovascular and microvascular complications have been associated with poor glycemic management indicated by higher levels of glycated hemoglobin (HbA1c) [4]. Chronic hyperglycemia is thought to directly impact myocardial stiffness and impaired relaxation and could contribute to LVDD [5]. Increased HbA1c levels have been associated with a decline in diastolic function in diabetic patients in several echocardiographic studies [6]. While some studies find weak or no correlation and others indicate a stronger relationship, the strength and nature of this association remain unclear [7]. Knowledge of this relationship is needed for early intervention in preventive care of persons with diabetes [8]. The purpose of this study was to examine the association between HbA1c and LVDD using Doppler echocardiography in patients with type 2 diabetes [9]. Therefore, it is of interest to study and further describe the association between HbA1c levels and left ventricular diastolic dysfunction in patients with type 2 diabetes, as this may provide valuable insights for early diagnosis and preventive strategies.

## Materials and Methods:

This cross-sectional research study was carried out in the Department of Medicine BA-9 Medical College's. Over the course of one year, 100 participants with known type 2 diabetes were enrolled for participation in this study. Inclusion criteria consisted of, age between 30 to 70 years, established diagnosis of type 2 diabetes with a duration of 1 year or more and willingness to take part in echocardiography. Patients with uncontrolled hypertension, valvular heart disease, or known coronary artery disease excluded. Demographic details along with clinical were recorded. Blood samples were drawn specifically for HbA1c estimation by high performance liquid chromatography. Diastolic parameters, which included deceleration time (DT), left atrial volume index (LAVI), E/A ratio and E/e', were evaluated using a transthoracic echocardiogram, which is the standard of care.

## Results:

One hundred T2DM patients, whose mean age was 55.2 ± 9.6 years, made up the study population. 58% of the study group was made up of men. The mean HbA1c was 8.3 ± 1.6%. Diastolic dysfunction was present in 66% of participants. Among these, Grade I dysfunction was the most prevalent. A statistically significant positive correlation (r = 0.62, p < 0.001) was observed between HbA1c levels and E/e' ratio, while an inverse correlation was noted between HbA1c and E/A ratio (r = -0.54, p < 0.01). LAVI and DT values were also significantly abnormal in patients with poor glycemic control. The prevalence of LVDD increased proportionally with higher HbA1c strata (>9%). Table 1 (see PDF) presents the baseline demographic and clinical characteristics of the study participants, showing that the mean age was 55.2 ± 9.6 years with a male predominance of 58%. The mean duration of diabetes was 7.4 ± 3.2 years and the average HbA1c was 8.3 ± 1.6%, indicating generally poor glycemic control in the study population. Table 2 (see PDF) shows the distribution of diastolic dysfunction grades, where 66% of patients exhibited LVDD, most commonly Grade I (40%), followed by Grade II (20%) and Grade III (6%), while 34% had normal function. Table 3 (see PDF) demonstrates the correlation between HbA1c and echocardiographic parameters, revealing that higher HbA1c values were significantly associated with an increased E/e' ratio and LAVI, prolonged DT and a reduced E/A ratio, confirming HbA1c as a strong predictor of impaired diastolic function. Table 4 (see PDF) presents the HbA1c group-wise comparison of LVDD prevalence, showing a stepwise increase from 25% in patients with HbA1c <7.0 to 63% in those with HbA1c 7.1-8.5 and 87% in those with HbA1c >8.5, indicating a clear dose-response relationship between poor glycemic control and worsening diastolic dysfunction.

## Discussion:

This study shows that in individuals with type 2 diabetes mellitus, glycemic control and left ventricular diastolic dysfunction are significantly correlated [10]. Our findings demonstrate that higher HbA1c levels are associated with worsened echocardiographic indices of diastolic function, corroborating previous studies [11]. Specifically, elevated HbA1c showed a positive correlation with E/e' ratio and LAVI and a negative correlation with E/A ratio, which are established markers of diastolic dysfunction [12]. Low-grade inflammation, oxidative stress and advanced glycation end products (AGEs) are all caused by hyperglycemia, which result in myocardial fibrosis and stiffness [13]. These processes affect relaxation and elevate filling pressures in the left ventricle [14]. The association of LVDD and poor glycemic control implies a dose-response effect, like seen in our group-wise comparison analysis [15]. The clinical implications are substantial. Diastolic dysfunction is a precursor to heart failure with preserved ejection fraction (HFpEF), which is increasingly recognized in diabetic patients [16]. Early identification of subclinical LVDD using echocardiography in high HbA1c individuals may allow for timely intervention through stricter glycemic control, lifestyle modification and pharmacological therapy [17]. However, our study is limited by its cross-sectional design, which precludes causality inference. Longitudinal studies are required to assess the impact of HbA1c reduction on the progression or regression of diastolic dysfunction [18]. Additionally, strain imaging or tissue Doppler indices might offer superior sensitivity for early detection [19]. It is well known that echocardiography has increased sensitivity in detecting LV dysfunction and hypertrophy. Diabetes patients with high glycosylated hemoglobin (HbA1c) levels have a larger LV mass. As blood sugar stability improves, the risk of LV hypertrophy decreases [20].

## Conclusion:

There exists a significant association between poor glycemic control and left ventricular diastolic dysfunction in patients with type 2 diabetes. Echocardiographic screening may be beneficial in diabetics with elevated HbA1c for early cardiac risk assessment and intervention.
